# Nutrition and Cancer Risk from the Viewpoint of the Intestinal Microbiome

**DOI:** 10.3390/nu13103326

**Published:** 2021-09-23

**Authors:** Yoshimu Tanaka, Shin Shimizu, Masahiko Shirotani, Kensho Yorozu, Kunihiro Kitamura, Masayuki Oehorumu, Yuichi Kawai, Yoshitaka Fukuzawa

**Affiliations:** 1 Jinzenkai Tanaka Clinic, 2-3-8, Ikunonishi, Ikuno-ku, Osaka 544-0024, Japan; 2 The Association for Clinical Research of Fecal Microbiota Transplantation Japan, 2-1-40, Katamachi, Miyakojima-ku, Osaka 534-0025, Japan; shin@shinbiosis.com (S.S.); lukesashiya@gmail.com (M.S.); info@yorozu-cl.com (K.Y.); kunihiro@kitamura.or.jp (K.K.); info@lifeclinic-t.jp (M.O.); info@kawai-medical.com (Y.K.); yofuku@aichi-med-u.ac.jp (Y.F.); 3 Symbiosis Research Institute, 6-7-4-106, Minatojimaminami-machi, Chuo-ku, Kobe, Hyogo 650-0047, Japan; 4 Luke’s Ashiya Clinic, 8-2, Ohara-cho, Ashiya, Hyogo 659-0092, Japan; 5 Ishinkai Yorozu Clinic, 1-118-4, Mihagino, Tottori 689-0202, Japan; 6 Kitamura Clinic, 4-3-8, Nishiki-machi, Onojo, Fukuoka 816-0935, Japan; 7 LIFE Clinic Tateshina, 3317-1, Toyohira, Chino, Nagano 391-0213, Japan; 8 Yuakai Kawai Clinic for Internal Medicine, 3-7-14, Higashi-Nakahama, Joto-ku, Osaka 536-0023, Japan; 9 Aichi Medical Preemptive and Integrative Medicine Center, Aichi Medical University Hospital, Yazakokarimata, Nagakute, Aichi 480-1103, Japan

**Keywords:** cancer, nutrition, intestinal microbiome, fecal microbiota transplantation, ultrafine bubble water, probiotics, prebiotics, symbiotics, immunity

## Abstract

There are various important factors in reducing the risk of cancer development and progression; these factors may correct an unbalanced intake of nutrients to maintain the living body’s homeostasis, detoxify toxic materials, acting as an external factor, and maintain and strengthen the body’s immune function. In a normal cell environment, nutrients, such as carbohydrates, lipids, proteins, vitamins, and minerals, are properly digested and absorbed into the body, and, as a result, an environment in which cancer can develop and progress is prevented. It is necessary to prevent toxic materials from entering the body and to detoxify poisons in the body. If these processes occur correctly, cells work normally, and genes cannot be damaged. The most important factor in the fight against cancer and prevention of the development and progression of cancer is the immune system. This requires a nutritional state in which the immune system works well, allowing the intestinal microbiome to carry out all of its roles. In order to grow intestinal microbiota, the consumption of prebiotics, such as organic vegetables, fruits, and dietary fiber, and probiotics of effective intestinal microbiota, such as fermented foods and supplements, is required. Symbiosis, in which these organisms work together, is an effective means of reducing the risk of cancer. In addition, fecal microbiota transplantation (FMT) using ultrafine bubble water, produced specially by the Association for Clinical Research of Fecal Microbiota Transplantation Japan, is also useful for improving the nutritional condition and reducing the risk of cancer.

## 1. Introduction

The initiation and development of cancer are related to the deterioration of the cellular environment as a result of an imbalance in nutrient intake and contact with toxic substances. Dysbiosis (the alteration and simplification of the intestinal microbiota composition) can induce abnormal functioning of the immune system and chronic inflammation, and can also cause carcinogenesis and the promotion of cancer processes. Therefore, it is necessary to recognize the importance of intestinal microbiota and of the improvement of the intestinal environment in the prevention of carcinogenesis and ongoing cancer processes.

Recently, metagenomics analysis through high-depth next-generation sequencing of bacterial 16S ribosomal RNA has enabled the identification and relative quantification of vast numbers of organisms in the intestinal microbiome. The human intestine contains approximately 1000 different species of known bacteria, with the largest number of bacteria (more than 100 trillion) equivalent in weight to the liver at 1.5 kg [1]. The number of genes in this microbiome is approximately a thousand times that of human gene content. The intestinal microbiome controls human metabolism, the production of nutrients, immune function, and communication with the other organs. The balance of intestinal microbiota is strongly linked to human diet, mental and physical stress, environment, and lifestyle, and this microbiome maintains a symbiotic relationship with the body. The failure of this symbiotic relationship induces various diseases, such as many types of cancer [2], diabetes mellitus, cardiovascular disease, autoimmune diseases, and neuropsychological diseases.

In the development and promotion of cancer, oral bacteria, particularly periodontal bacteria [3], are closely connected with intestinal microbiota, considering the involvement of *Fusobacterium nucleatum* in colorectal and esophageal cancer [4,5] and *Neisseria enlogata* and *Streptococcus mitis* in pancreatic cancer [6]. The carcinogenesis of hepatic cancer is related to *Clostridium cluster XI* and *XIVa* in a high-fat diet [7,8,9].

In this review, we focus on the prevention of various cancers from the viewpoint of the intestinal microbiome. We then introduce clinical studies on the use of prebiotics, probiotics, and FMT (especially our method using ultrafine bubble water) with nutrition [10,11,12,13,14,15,16,17,18,19].

## 2. Mechanism of Onset and Progression of Cancer

**Figure 1:**
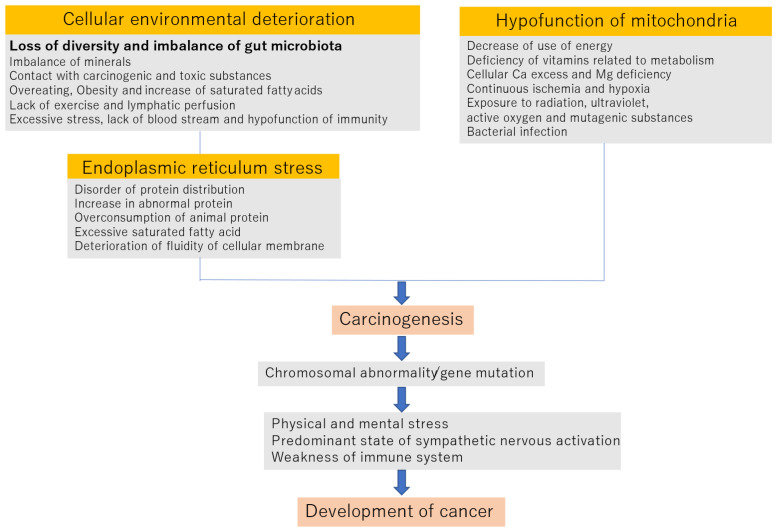
Mechanism of carcinogenesis and development of cancer.

Figure 1 shows that the onset and progression of cancers cause worsening of the cellular environment.

### 2.1. Deterioration of the Cellular Environment

Imbalance of minerals: The blood concentrations of zinc, magnesium, selenium, iron, manganese, and molybdenum are lower in cancer patients. Conversely, the concentrations of copper, aluminum, and lead are increased in cancer patients [20].

Exposure to carcinogens and toxic substances: Exposure to mold aflatoxin, tobacco tar, heavy metals, such as asbestos and mercury, and endogenous carcinogens, such as sex hormones, reduces the ability to scavenge active oxygen [21].

Overeating and obesity: Overeating reduces the control of carbohydrates and fat metabolism and relatively increases the levels of saturated fat. As a result, it becomes a trigger of chronic inflammation [22].

Lack of exercise and the lowering of lymph flow: The promotion of chronic inflammation occurs when toxic and metabolic substances stay in the body for long periods [23].

Lack of diversity in intestinal microbiota is related to an increased production of toxic substances from the overconsumption of high-protein and high-fat diets such as meats. Undecomposed toxic substances, the proliferation of harmful bacteria, and the breakdown of the balance of intestinal microbiota induce abnormalities within the immune system.

### 2.2. Endoplasmic Reticulum STRESS

The excess accumulation of protein, particularly animal protein, an increase in abnormal protein, and the deterioration of the fluidity of the endoplasmic reticulum membrane due to excessive saturated fat induce endoplasmic stress.

In such cases, the endoplasmic reticulum may be processed by autophagy to avoid stress. However, if endoplasmic reticulum stress is not relieved, cells are programmed to undergo apoptosis, which is executed through commands from mitochondria. However, when mitochondrial dysfunction occurs, autophagy and apoptosis do not occur properly, and cells become cancerous [24,25,26,27].

### 2.3. Hypofunction of Mitochondria

Mitochondrial dysfunction—that is, mitochondrial aging—may be caused by a decrease in energy utilization, a lack of vitamins related to metabolism, an excess of cellular calcium and a lack of cellular magnesium, continuous ischemia and hypoxia, radiation exposure, ultraviolet superoxide and mutagenic substances, and infection by bacteria such as *Helicobacter pylori*.

Much of the body’s active oxygen is generated from aged mitochondria, which damages intracellular proteins, changes the lipid portion of phospholipids, which are cell constituents, into lipid peroxides and damages DNA. If such a state continues, cell aging will progress, and cancer will develop [28,29,30].

When the above three factors occur for an extended period of time, they can cause carcinogenesis and induce chromosomal abnormality and gene mutation. After this, if the immune function is normal, cancer cells induce apoptosis via attack from cytotoxic T lymphocytes and natural killer cells.

It is also known that the sympathetic/parasympathetic nervous system, associated with mental and physical stress, is involved in carcinogenesis. It is speculated that changes in lymphocyte dynamics associated with continuous excitement of the sympathetic nervous system due to chronic stress act to weaken the acquired immune response. That is, when the sympathetic nerve is excited, effector T cells cannot move from the lymph nodes to the tumor tissue and cannot participate in the process of eliminating tumor cells. Furthermore, stimulation of the β2 adrenaline receptors expressed on dendritic cells (tumor-antigen-presenting cells for T cells) reduces the cells’ antigen-presenting ability and the cytokine-producing ability, resulting in dendritic cells with high sympathetic nervous system activity. Therefore, it is thought that the activation of T cells by dendritic cells is impaired. If the sympathetic nerve systems are exposed to long-term excessive mental stress, cancer develops due to immune hypofunction, such as decreased lymphocytes [31,32,33,34,35,36].

Chronic inflammation is caused by an imbalance of nutrients, and the intake and accumulation of toxic substances and abnormality in the immune system are the most important factors in carcinogenesis and the progression of cancer mechanisms. The intestinal microbiome is connected to all of these mechanisms. Therefore, the optimization of microbiota is important in preventing the onset and development of cancer.

## 3. The Role of the Intestinal Microbiome

The diversity and compositional balance of the intestinal microbiome are important to human health, and they change depending on factors, such as diet, age, living environment, and physical and mental stress (dysbiosis). Additionally, the intestinal microbiome is involved in the development and progression of cancer.

The main functions of the intestinal microbiome are metabolic control, production of useful materials, immune control, and communication with other organs. In particular, immune control is the most important in carcinogenesis.

### 3.1. Control of the Metabolism

Carbohydrate metabolism [37], lipid metabolism [38], and decomposition of dietary fiber [39] are performed by the intestinal microbiota. In particular, in carbohydrate metabolism, polysaccharides and oligosaccharides are absorbed partly in the small intestine as glucose by digestive enzymes. The remaining carbohydrates are digested by the enzymes of the intestinal microbiota and become the energy source of bacteria via anaerobic metabolism. Finally, organic acids or short-chain fatty acids (SCFAs), which are metabolic substances of the microbiota, are absorbed and used in the large intestine [40,41,42].

### 3.2. Production of Valuable Substances

Intestinal microbiota produce organic acids, such as SCFAs (butyric acid, propionic acid, and acetic acid), equol (from daidzein, a kind of soybean isoflavone) [43], ingredients of neurotransmitters (such as dopamine and serotonin) [44,45,46], various vitamins [47], and intravital hydrogen (*Ruminococcus*, *Roseburia*, *Clostridium*, and *Bacteroides*) [48].

In particular, the SCFA butyric acid is an energy source of colon epithelial cells, producing ATP in those cells. Butyric acid suppresses dysbiosis via the provision of an anaerobic environment to obligate anaerobes. Moreover, it is difficult in this environment to produce harmful secondary bile acid, making the surroundings in the colon weakly acidic and working to protect against colorectal cancer. Furthermore, butyric acid has the function of promotion of the redifferentiation from cancer cells to normal tissue cells by accelerating apoptosis, as well as having the ability to cure colorectal cancer. It has been reported that propionic acid suppresses the proliferation of hepatic cancer cells via the SCFA receptor hepatic cancer cells. Additionally, SCFA is related to the production of regulatory T cells, the differentiation of the anti-inflammatory M2 macrophage, and antibody formation by B cells. It can be estimated that the formation of cancer is connected with chronic inflammation, and digestible dietary fiber is hardly required in the production of SCFAs [49,50].

### 3.3. Control of Immunity

The intestinal microbiota in intestinal epithelial cells work to support the immune cells (regulatory T cells: CD4 + CD25 + Foxp3, Th17, and IgA) in the lamina propria. For example, *Bacteroides fragilis* induces the differentiation of Th17 and is related to the onset of colorectal cancer by way of the tumor proliferation of IL-22. Moreover, the differentiation of regulatory T cells by *Clostoridium* induces immune tolerance for the suppression of autoimmune diseases and inflammatory bowel diseases, and at the same time inhibits the attack against cancer by cytotoxic T cells [51].

### 3.4. Communication with Other Organs

Gut microbiota communicate with other organs, e.g., brain–gut interaction [52,53], gut–kidney linkage [54], and insulin secretion in the pancreas [55].

Homeostasis is maintained by activating the hypothalamic–pituitary–adrenal (HPA) axis and sympathetic nervous system when exposed to harmful stress. It has been reported that the intestinal flora affect these biological reactions, and it has become clear that signal transduction is performed via the microbiota–gut–brain axis [56].

D-amino acid (D-serine), produced by intestinal bacteria, has a nephroprotective effect [57,58]. Dietary phosphatidylcholine is converted to trimethylamine by intestinal bacteria and metabolized to trimethylamine oxide in the liver, making it a toxic substance for the kidneys, heart, and blood vessels [59].

## 4. Enterotype

Intestinal microbiota are broadly classified into three categories by their effect in daily dietary life, classified by the balance of nutrient intake, such as carbohydrates, fat, protein, and dietary fiber [60].

Type 1 (B1 and B2): *Bacteroides* is dominant. This type is related to the secretion of SCFAs, the prevention of obesity, and the onset of carcinoma scapegoating opportunistically according to the intestinal environment.

Type 2 (P): *Prevotella* is dominant. This type has a strong dietary fiber-degrading enzyme and is related to the habit of eating more starch and dietary fiber. It raises the risk of the onset of cardiovascular diseases.

Type 3 (R): *Ruminococcus* is dominant. This type accelerates the absorption of carbohydrates and the accumulation of fat and induces obesity as a result. It raises the risk of cardiovascular diseases, such as cerebral infarction and myocardial infarction.

The main intestinal microbiota associated with cancer are as follows:

(1) *Bifidobacterium*: This genus has catalytic activity of cyclic lactic acid and the effect of the prevention of cancer development. It neutralizes waste products, such as bile acid, and is immunity-strengthening, aids mental stability, and complements the activity of other microbiota in carbohydrate and lipid metabolism [61].
(2) *Lactobacillus*: This genus has catalytic activity of cyclic lactic acid and the effect of prevention of cancer development. It controls gene restoration and cell regeneration, is immunity-strengthening, aids mental stability, and complements the activity of other microbiota in carbohydrate and lipid metabolism [62].
(3) *Clostridium*: This genus promotes the secretion of regulatory T cells and has useful effects on allergic diseases, autoimmune diseases, and chronic inflammatory diseases via the secretion of SCFAs [63].
  *Cluster XVIII*: Involved in suppression of carcinogenesis and overreaction of the immune system.
  *Cluster XV*: Involved in activation of macrophages and induction of apoptosis.
  *Cluster IX* Involved in gene restoration and induction of apoptosis.
  *Cluster IV*: Involved in induction of regulatory T cells.
(4) *Akkermansia muciniphila*: Involved in regulation of the effect of immune checkpoint inhibitors [64,65].
(5) *Clostridium cluster Blautia*: Involved in the restoration of inflammatory tissues and mutated cells, and control of immunity [66].
(6) *Faecalibacterium prausnitzii*: Involved in suppression of obesity and the onset of diabetes mellitus, and in induction of regulatory T cells via the production of butyric acid [67].
(7) *Clostridium butyricum*: Involved in prevention of diseases via abnormal proliferation of resistant microbes, such as *Clostridium difficile*. It is a butyric acid-producing bacterium [68].

## 5. Improvement of Dysbiosis

In order to prevent the onset and development of cancer, it is important to control intestinal microbiota. Prebiotics, probiotics, and symbiotics that integrate these can be used to support the proliferation of useful microbiota and the suppression of harmful microbiota. However, if dysbiosis does not improve, a fecal microbiota transplantation (FMT) from a healthy donor patient should be performed.

### 5.1. Prebiotics

Prebiotics are hardly digestible food ingredients that have a beneficial effect on the host by selectively multiplying useful bacteria in the intestine and suppressing harmful bacteria [69]. Additionally, they are not hydrolyzed or absorbed in the upper part of the digestive tract. A prebiotic is a selective substrate for one or a limited number of beneficial bacteria (such as *Bifidobacterium*) that coexist in the large intestine, promoting the growth of those bacteria or activating their metabolism. The intestinal microbiota of the large intestine can be modified to favor a healthy composition, inducing systemic effects that are beneficial to the health of the host. Dietary fiber can be categorized as water-soluble or -insoluble [70,71,72,73,74,75], with the former including pectin (contained in fruits, potatoes, and vegetables), alginic acid (seaweeds), gums (soybeans, barley, and limewood), and glucomannan (konjac), and the latter including cellulose (soybeans, gobo, wheat bran, grains, and beans), hemicellulose (wheat bran, soybeans, grains, and gobo), lignin (wheat bran, grains, vegetables, beans, and cocoa), and chitin (classified as a kind of shell, including mushrooms). Indigestible oligosaccharides, such as fructooligosaccharide (FOS) [76] and galactooligosaccharide (GOS) [77], also act to maintain intestinal immunity by promoting IgA secretion into the gastrointestinal tract. α-Glucan, which is one of the glucose polymers, is digested by digestive enzymes in saliva and pancreatic juices and is ultimately decomposed into glucose and maltose, which are then absorbed in the small intestine and can be used as an energy source. However, these dietary fibers do not only adsorb and remove harmful substances; some examples of their other roles include benefiting the host by synthesizing and supplying vitamins and β-glucan, such as cellulose, as well as metabolism of indigestible carbohydrates by the indigestible bacteria that live in the colon and ensuring their bioavailability. In addition, SCFAs, which are tumor metabolites, act on the nervous system, the endocrine system, the immune system, etc., through various physiological actions, such as ligand activity and enzyme inhibitory activity, contributing to biological defense and metabolic homeostasis. SCFAs create an intestinal tight junction protective effect and also prevent the generation of inflammation-inducing substances, such as lipopolysaccharides (LPS) and toxic substances and harmful bacteria invasion [78]. Additionally, glucobrassicin from cruciferous vegetables (such as broccoli and cauliflower) is hydrolyzed, and indole-3-carbinol is produced. This is converted to diindolylmethane (DIM) by intestinal bacteria, which suppresses carcinogenesis and proliferation of cancer cells, and causes suppression of infiltration and metastasis [79]. DIM inhibits colon cancer by activating AhR (aryl hydrocarbon receptor), as well as inhibiting breast cancer, ovarian cancer, uterine cancer, and lung adenocarcinoma by normalizing the sex hormone metabolism [80,81]. Glycoside sugars from umbelliferae vegetables (carrots, celery, and parsley) remove sugars via intestinal bacteria and become apigenin and luteolin, which have anticancer effects.

Promotion of the production of hydrogen in the large intestine by indigestible sugars such as dietary fiber contributes to a reduction in oxidative stress and the suppression of oxidative damage.

In an unpublished study, we measured the urinary indoxyl sulfate (indican) concentration by giving capsules containing an Si-based agent [82,83] and dietary fiber to six cancer patients for one month and estimated how the intestinal environment was changed (Table 1).

Indole is synthesized from dietary-protein-derived tryptophan in the intestine by dysbiotic bacteria, converted to indoxyl sulfate (indican) in the liver, and then excreted in the urine, and dysbiosis can be evaluated by measuring it [84,85].

In addition, the Si-based agent continuously produced hydrogen in the gastrointestinal tract for 24 h or more (Institute of Scientific and Industrial Research, Osaka University, Osaka, Japan).

Four out of the six cancer patients showed an improvement in their intestinal environment for the short period of one month.

### 5.2. Probiotics

Probiotics refer to living microorganisms that produce healthy beneficial effects for the host by improving the flora balance in the intestine when administered in adequate amounts.

Sufficient safety is guaranteed. Probiotics are originally members of the intestinal flora, able to withstand gastric juice, bile, etc., and to reach the intestines alive. Probiotics can adhere to the insides of the intestines and grow while maintaining the form of food for an effective number of bacteria. They are easy to handle and are inexpensive. Probiotics need to meet these previous requirements to be considered effective. Examples of probiotics include supplements such as yogurt, natto, miso, lactic acid bacteria, and bifidobacteria. They are often used in diseases, such as infectious diarrhea (travelers’ diarrhea), antibiotic-associated diarrhea, *Helicobacter pylori* infection, constipation, irritable bowel syndrome, hepatic encephalopathy, Crohn’s disease, and ulcerative colitis [86,87,88,89].

**Figure 2:**
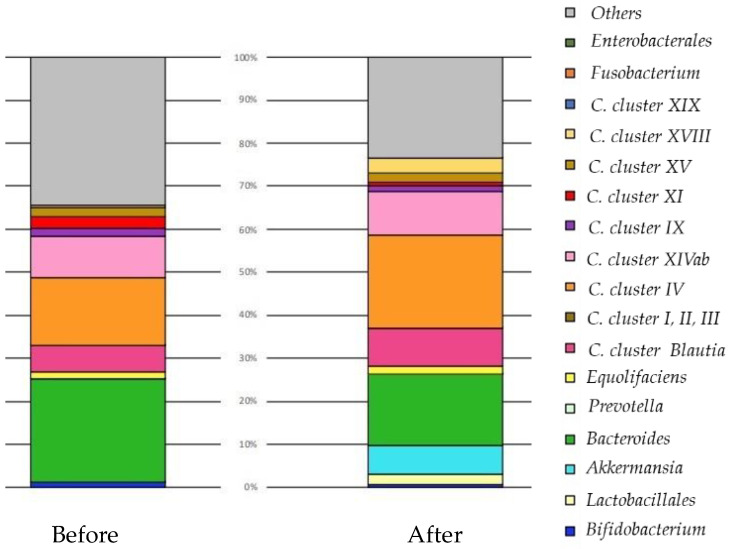
The change in the balance of intestinal microbiota by original profiling of 16SrRNA sequencing before and after the oral administration of probiotic capsules over three months. Forty-seven years old, female, breast cancer (left C region), T2N3aM0, stage IIIc, ER100% PgR40% HR2(3+) Ki67:50%. She took encapsulated *Lactobacillus* supplements for approximately three months. No adverse events were observed while taking encapsulated *Lactobacillus* supplements. After FMT, the diversity of intestinal bacteria increased. *Akkermansia* proliferated and immune cells may have been fortified. *Lactobacillus* increased, and may have progressed tissue repair and reconstructed tissue after destroying breast cancer. The size of her breast cancer remained unchanged, so she agreed to receive standard treatment. One year after the operation, she is in good general condition and is receiving hormone therapy. *Clostridia (C)*.

We present a case of a cancer patient that confirms the effect of a probiotic supplement in which six lactic acid bacteria were combined (Figure 2).

#### 5.2.1. Background

She presented to our clinic and chose not to receive standard treatment (surgery, anticancer agent, or radiation therapy) right away. We convinced her that standard treatment was the best option for treating her condition. However, she said that she would accept standard treatment if she did not improve after taking probiotics for three months.

#### 5.2.2. Materials and Methods

She signed an informed consent form before the treatment.

She took encapsulated Lactobacillus supplements (Lactobacillus helveticus, Lactobacillus reuteri, Lactobacillus fermentum, Lactobacillus delbrueckii, Lactobacillus rhamnosus, and Lactobacillus plantarum) for approximately three months, after which her intestinal flora balance, immune function, etc., were tested. It was recommended that milk and dairy products be avoided and that a diet of brown rice, soy foods, organic vegetables, and dietary fiber be consumed.

#### 5.2.3. Results

Before: There were more bacteria (*Clostridia cluster XI*) promoting inflammation to boost immunity. *Clostridia cluster IV* increases immune tolerance so that it does not come under attack itself. *Clostridia* *Blautia* is aggressively powerful, and *Clostridia cluster IV* and *Equolifaciens* (equol-producing bacteria) repair tissue. There was almost no *Lactobacillus*, and immunity could not be guaranteed as a whole. By increasing the number of *Lactobacillus*, inflammation was suppressed, and the balance with Treg (suppressive T cells) improved. There was almost no *Prevotella*, and increasing the number of *lactobacilli* improved the glucose metabolism.

After: Diversity increased. *Akkermansia* proliferated and immune cells may have been fortified. In addition, *Lactobacillus* increased and may have progressed tissue repair and reconstructed tissue after destroying breast cancer. If *Lactobacillus* + *Bifidobacterium* + *Akkermansia* increase to 15–20% (50% with *Bacteroides* added), the cancer may become smaller.

In fluorescence-activated cell sorting after three months, the proportion of regulatory T lymphocytes had decreased, the cytotoxic T lymphocyte/regulatory T lymphocyte ratio increased, and the aggression toward cancer increased.

No adverse events were observed while taking encapsulated *Lactobacillus* supplements.

Three months later, her intestinal flora had improved. Clinically, however, the size of her breast cancer remained unchanged, so she agreed to receive standard treatment. One year after the operation, she is in good general condition and is receiving hormone therapy.

### 5.3. Fecal Microbiota Transplantation (FMT)

The effects of prebiotics, probiotics, or symbiotics in severe cases are uncertain. FMT is a treatment method that enables dramatic changes in the intestinal environment and exerts a therapeutic effect. FMT is a method of administering the intestinal microorganisms contained in a healthy person’s stool to a patient with a disease. FMT has been shown to be useful in many diseases, such as relapsed and refractory *Clostridium difficile* infection (CDI), irritable bowel syndrome (IBS), inflammatory bowel disease, such as ulcerative colitis, autism spectrum (ASD), neuropsychiatric issues, such as depression, and allergic conditions, such as atopic dermatitis. Furthermore, management with FMT has also been used to treat the onset and progression of cancer, which is associated with immune disorders and chronic inflammation [90,91,92,93,94,95,96,97].

We performed FMT using characteristic methods: administration method, administration route, preparation of a bacterial solution, number of administrations, and selection of donors were conducted differently from standard protocol. Without using an endoscope, 250 mL of donor feces dissolved in a special bacterial solution was directly injected into the rectum via a catheter enema. The injection was completed in approximately 5–10 min. After this, the posture was changed, and vital signs before and after injection were observed. To date, we have treated about 400 patients, and in each case, the treatment was completed without any side effects. The stool used for transplantation was a safe stool from a healthy volunteer donor enrolled in Japanbiome who had passed a strict examination. Regarding the method for adjusting the bacterial solution, physiological saline is often used, but in previous work by our study group, NanoGAS^®^ water (WIPO: WO/2019/168034) was developed by combining a rotary shear method with physical shearing by a hollow thread filter and then applying a magnetic field to perform electrical rectification. Ultrafine bubble water was used to enhance the colonization of bacteria [98].

**Figure 3:**
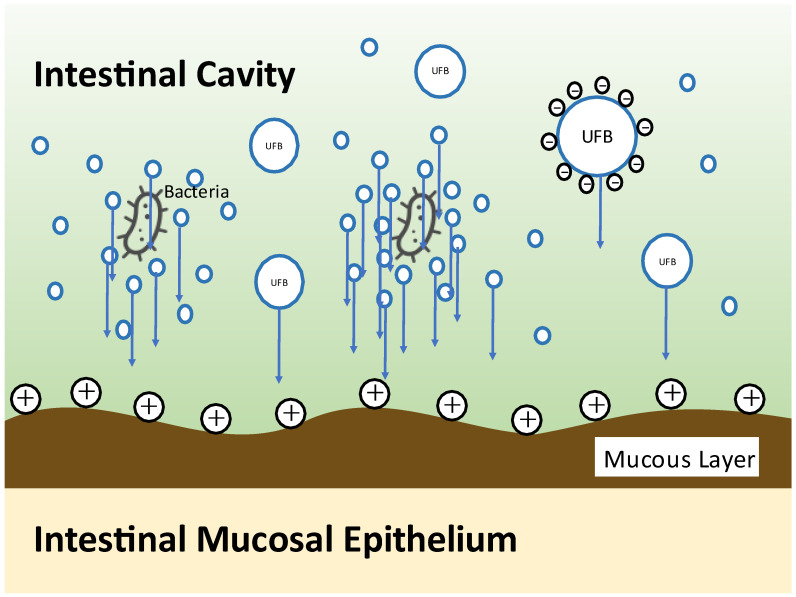
Illustration of bacterial engraftment by NanoGAS^®^ water.

**Figure 4:**
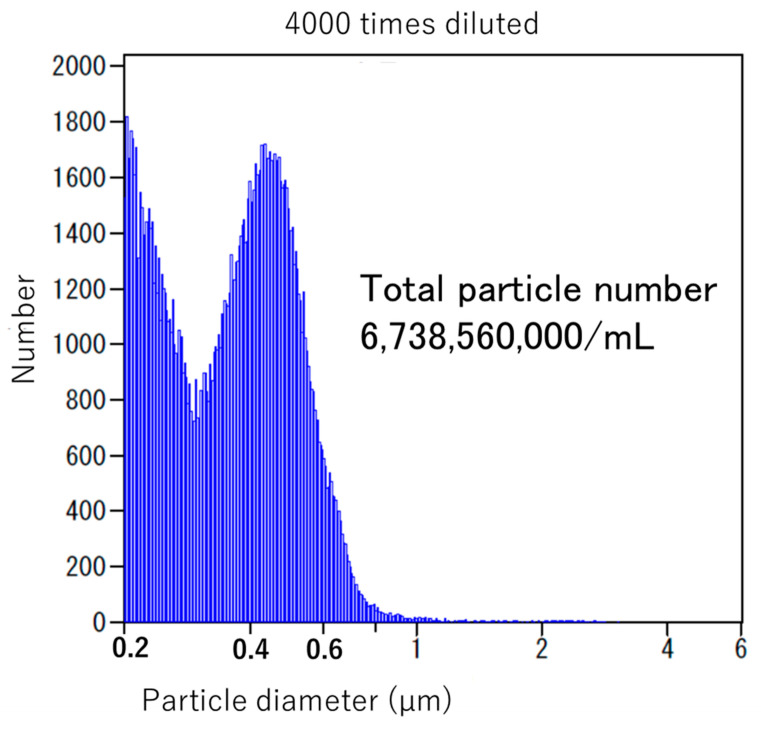
Particle number of NanoGAS^®^ water.

NanoGAS^®^ water is negatively charged. The bacteria cannot come into contact with one another, and the bacteria do not overgrow. It also inhibits the formation of biofilms, so it is an extremely useful bacterial adjusting solution (Figure 3). Figure 4 shows that it was measured using an instrument from Beckman Coulter, Inc. (Brea, CA, USA). There were 6,738,560,000 particles in 1 mL, and particles smaller than 0.2 μm were stable for a long period of time.

**Figure 5:**
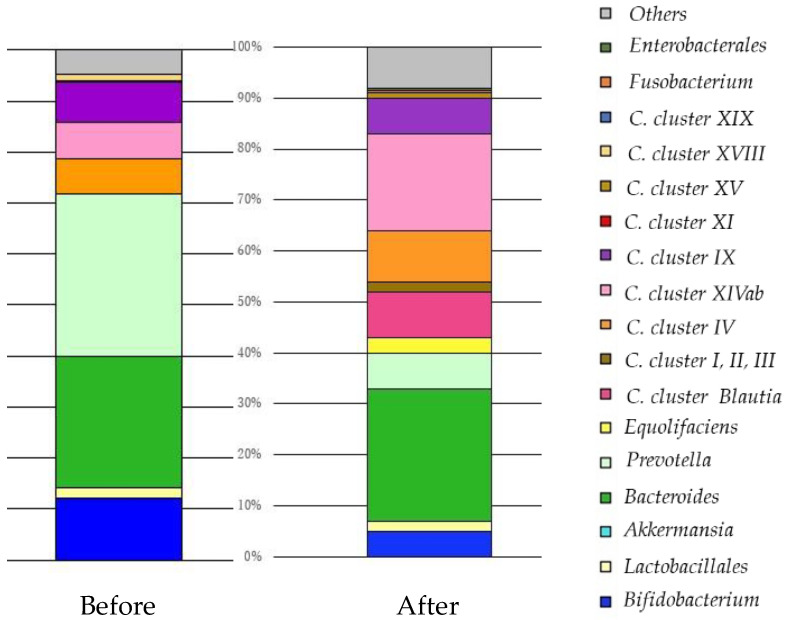
The change in the balance of intestinal microbiota by original profiling of 16SrRNA sequencing before and after FMT in six months. Sixty-six years old, male, lung adenocarcinoma, Stage IV. He received FMT using the bacterial solution adjusted with NanoGAS^®^ water once a week for six months. No adverse events were observed during the FMT procedure. After FMT, the diversity of intestinal bacteria increased, *cluster IX:XIVa* improved to 1:2, and immunity was reinforced, resulting in acquisition of tissue repair ability, improvement of glucose, and lipid metabolism. As a result, his QoL and prognosis improved. He survived approximately one year after six months of FMT. *Clostridia (C.)*.

We present a case of a cancer patient that confirms the effect of FMT using the bacterial solution adjusted with NanoGAS^®^ water (Figure 5).

#### 5.3.1. Background

He had received no anticancer drug treatment, nor had he received any other treatment, such as immunotherapy. His general condition was so poor that he was advised to move to palliative care (best supportive care). Before FMT treatment, he had difficulty walking independently and had difficulty breathing, so he wore an oxygen mask and continued to inhale oxygen.

#### 5.3.2. Materials and Methods

He was signed an informed consent form before the practice.

The bacterial solution for FMT was adjusted with NanoGAS^®^ water. He received FMT once a week for six months, and an intestinal flora balance test was performed before and after FMT.

#### 5.3.3. Result

Before: *C. cluster IX:XIVa* was 1:1, and immunity was weakened. The parasympathetic nervous system was dominant. *Prevotella* abnormally proliferated, and it was used as energy for tumor cells with a high sugar metabolism.

After: *C. cluster IX:XIVa* improved to 1:2 and immunity was reinforced, resulting in acquisition of tissue repair ability, improvement of glucose, and lipid metabolism.

No adverse events were observed during the FMT procedure.

After FMT, he was able to walk on his own and demonstrated improved appetite and improved QoL. He was told by his doctor that he had a prognosis of three months, but he survived approximately one year after six months of FMT.

## 6. Discussion

Chronic inflammation as a result of deterioration of the cellular environment due to nutrient intake and the accompanying imbalance of immune function is deeply involved in the onset and progression of cancer. At the same time, the intestinal flora, which has a symbiotic relationship with humans, is deeply involved in the chronic inflammation. When a positive symbiotic relationship breaks down, various chronic diseases develop, such as cancer, inflammatory bowel disease (ulcerative colitis and Crohn’s disease), autoimmune disease, allergic disease, neuropsychiatric disease (autism, depression, ADHD, and dementia), arteriosclerotic disease, diabetes mellitus, and various chronic diseases, such as chronic kidney disease, obesity, and NAFLD. In this review, we discuss the deep involvement of nutrients and intestinal bacteria in the development and progression of cancer [99,100]. In order to improve outcomes, we introduced prebiotics that support nutrient intake, probiotics that contain intestinal bacteria themselves, and FMT. In particular, our FMT process is a method of increasing the colonization of the transplanted patient using micro-nano bubble (ultrafine bubble) water (NanoGAS^®^ water). Conventional intestinal flora transplantation techniques (with saline) are thought to require several days for the transplanted microorganisms derived from a living body to colonize in a live state in another living body. Accordingly, it is thought that most of the transplanted intestinal flora is excreted over those several days; as a result, this causes a lack of retention of the transplanted intestinal flora in the body of the patient. Moreover, it has been reported that the colonization ratio (engraft ratio) of the microorganisms derived from a living body after transplantation is no more than 20–30% despite various attempts. As a result, the effect on the final goal of the treatment of diseases is not as great as expected. We developed an apparatus for generating nanobubbles with a diameter smaller than 1 μm (NanoGAS^®^). By generating a bacterial solution using NanoGAS^®^ water, we improved the technology for engrafting bacteria. FMT is easily performed during outpatient treatment via the catheter enema method without using an endoscope. Fecal microorganisms can be wrapped in bubbles and guided to the inner mucous layer, making it easier for them to engraft. We implemented this method with 347 patients, and the improvement rate was 77.8%. Although the study had a small number of cancer patients (19 cases), improvement of symptoms, QoL, and prognosis were observed following FMT.

Approximately 1700 years ago, Ge Hong recorded the use of feces to treat food poisoning and severe diarrhea. The Human Microbiome Project, which was started in the USA in 2007, is being promoted as a joint project between the USA, EU, Japan, and China and is attracting attention similar to the Human Genome Project. In 2013, FMT became used for *Clostridium difficile* infectious enteritis, and applied research for its use in other diseases has begun. The precision medicine initiative (PMI) that began in the USA in 2015 provided momentum and, with the progress of technological development for practical use, FMT is currently positioned as a “pharmaceutical product consisting of bacterial preparations.” Expectations are rising for early approval as a “first-in-class.” In the USA, it has already been developed and sold as an enema preparation, an oral preparation, and an oral capsule preparation by the NPO “OpenBiome” established in 2013. More recently, technological improvements in the development of bacterial transplantation without using feces have been made. Strain consortia consisting of multiple strains (2–12 strains), single strains, synthetic molecules, such as glycans, that change the distribution of bacterial flora, bacterial secretions, and selective elimination of target bacteria, have begun to be developed as next-generation drugs for bacterial preparations. Approximately 13% of the projects under consideration using the current bacterial preparations are in the field of oncology [101,102].

In the future, nutrition-based intestinal microbiota diseases such as cancer will be studied in association, and it is expected that treatment options for diseases such as cancer will be expanded by treatment methods that control intestinal microbiota.

## Figures and Tables

**Table 1:** The change in urinary indoxyl sulfate concentration (μM/g creatinine) after oral administration of capsules containing a Si-based agent and water-soluble dietary fiber in cancer patients after one month.

	Profile (Age, Sex, Cancer, Stage)	Before (μM/g Creatinine)	After (μM/g Creatinine)
1	42, F, ovarian, I	333	278
2	70, F, breast, II	622	489
3	73, M, oropharyngeal, III	326	482
4	58, F, colorectal, III	350	180
5	50, F, melanoma, III	405	367
6	43, F, breast, III	247	350
